# Computational Modeling of PI3K/AKT Pathway in Bipolar Disorder and Type 2 Diabetes: Implications for Lithium Treatment and Curcumin as a Potential Alternative

**DOI:** 10.3390/ijms262010026

**Published:** 2025-10-15

**Authors:** Jing Li, Wenqing Wang, Yajunzi Wang, Yang Hao, Lei Fu, Xin Liu

**Affiliations:** 1 Wisdom Lake Academy of Pharmacy, Xi’an Jiaotong-Liverpool University, Suzhou 215123, China; lj@ism.cams.cn (J.L.); wenqing.wang21@alumni.xjtlu.edu.cn (W.W.); yajunzi.wang17@student.xjtlu.edu.cn (Y.W.); haoyang25@sjtu.edu.cn (Y.H.); lei.fu@xjtlu.edu.cn (L.F.); 2 Institute of Systems, Molecular and Integrative Biology, University of Liverpool, Liverpool L69 7ZX, UK

**Keywords:** bipolar disorder, type 2 diabetes, PI3K/AKT pathway, computational modeling, GSK3β inhibition

## Abstract

Bipolar disorder (BD) exhibits a high comorbidity rate with type 2 diabetes (T2D), suggesting shared pathophysiological mechanisms. Although lithium serves as the first-line treatment for BD, its underlying therapeutic mechanism and potential effects on T2D remain incompletely understood. This study identified the PI3K/AKT pathway as a key link between these disorders. Using an ordinary differential equation (ODE) model that integrates the PI3K/AKT pathway with the phosphatidylinositol (PI) cycle, we simulated lithium’s regulatory effects in BD treatment. Our simulations revealed that lithium’s primary inhibitory effect on GSK3β stems from direct binding, which restores insulin sensitivity, suggesting potential benefits for both BD and T2D, particularly in their comorbid state. Additionally, molecular docking studies indicated that curcumin, a potentially safer alternative to lithium, exhibits enhanced anti-inflammatory properties by binding to both GSK3β and p38 MAPK. These findings provide novel insights into the molecular mechanisms connecting BD and T2D and propose new therapeutic strategies for their management.

## 1. Introduction

Bipolar disorder (BD) is a complex mental health condition characterized by recurrent, alternating episodes of mania (or hypomania) and depression, accompanied by significant mood dysregulation and functional impairment [1,2,3]. Clinically, manic episodes present with elevated or irritable mood, increased energy, reduced need for sleep, and impaired judgment, while depressive episodes manifest with persistent low mood, anhedonia, fatigue, and cognitive difficulties. Type 2 diabetes (T2D) is a chronic metabolic disorder characterized by insulin resistance and progressive pancreatic β-cell dysfunction, resulting in chronic hyperglycemia [4,5]. The clinical presentation of T2D includes elevated fasting glucose levels, increased HbA1c, and associated complications such as cardiovascular disease, neuropathy, and nephropathy [5,6].

Clinical observation and epidemiological studies clearly indicate that individuals with BD face a disproportionately high prevalence of T2D compared to the general population [7,8], posing a significant clinical challenge. This high comorbidity is associated with a more chronic illness course, treatment resistance, more severe functional impairment, and significantly increased mortality [9]. Within this comorbid population, metabolic disturbances, particularly impaired glucose metabolism, are especially common [10], posing a serious threat to patients’ long-term prognosis and highlighting the urgent importance of investigating the underlying shared pathophysiological mechanisms.

This clinical challenge points to a core scientific question: why are a psychiatric disorder and a metabolic disease so closely intertwined? Although insulin resistance is a key link, growing evidence suggests that the profound biological connection between the two is rooted in a complex network of multi-system dysregulation that extends beyond a single mechanism. In addition to abnormal energy metabolism, these shared pathophysiological pillars include chronic inflammation [8], hypothalamic–pituitary–adrenal (HPA) axis dysfunction [11], and abnormalities in calcium signaling and apoptosis pathways [12].

Chronic low-grade inflammation is a fundamental pathological state shared by BD and T2D [8]. In both disorders, elevated levels of pro-inflammatory cytokines (such as TNF-α and IL-6) are observed, which exacerbate peripheral insulin resistance in T2D and manifest as neuroinflammation in BD, impairing neural plasticity [10]. Hyperactivity of the HPA axis is another key link [11]. One of the characteristics of BD is a chronic elevation in cortisol (hypercortisolism), and cortisol directly antagonizes the action of insulin and promotes gluconeogenesis, thereby directly translating psychological stress into metabolic dysregulation [11]. Furthermore, mitochondrial dysfunction and oxidative stress are also common features of both diseases, leading to insufficient cellular energy supply and overproduction of reactive oxygen species (ROS); this damages neurons in BD and impairs pancreatic β-cell function in T2D [13,14,15]. Finally, dysregulation of calcium signaling homeostasis, partly stemming from major genetic risk genes for BD (such as CACNA1C), can lead to mitochondrial calcium overload, which in turn triggers apoptosis [16,17]. This process is not only related to neuronal loss in BD but is also a key mechanism of pancreatic β-cell death in T2D [18]. Recent evidence has established that the PI3K/AKT signaling pathway serves as a shared pathophysiological mechanism between T2D and neuropsychiatric disorders (NPDs), including BD [12], providing a molecular framework for understanding their frequent comorbidity.

These seemingly independent pathways frequently converge on the PI3K/AKT signaling pathway, making it a central hub for inflammatory, stress, and cell survival signals [19]. As a key downstream pathway of insulin signaling, its activation begins when insulin binds to its receptor, triggering a series of phosphorylation cascades that ultimately activate AKT. Activated AKT then inactivates its downstream targets, including GSK3β, through phosphorylation on its N-terminal [20]. In the pathological state of BD, the PI3K/AKT signaling pathway is thought to be dysfunctional, leading to the chronic overactivation of GSK3β. This overactivation is not only a key element of insulin resistance in T2D [21] but also disrupts neuronal energy metabolism and subsequent mitochondrial homeostasis in BD, making it a “pathological convergence point” linking the two diseases [12,22].

Alongside the dysregulation of GSK3β, a second major theory, the “inositol depletion hypothesis”, points to the phosphatidylinositol (PI) cycle as another core element of BD pathology [23]. The PI cycle is a vital second messenger system, and its dysregulation in BD is evidenced by abnormal concentrations of its key component, myo-inositol, in the brains of patients [24,25,26]. Specifically, manic states are often associated with elevated myo-inositol levels, while depressive states are linked to decreased levels, suggesting that disruptions in this cycle are fundamental to the mood instability characteristic of the disorder [23,27,28]. Therefore, to construct a model that more accurately reflects the complex cellular environment of BD, this study integrates the PI cycle pathway with the PI3K/AKT pathway. This integrated model connects the two pathways through the shared molecule PIP2, aiming to more comprehensively capture the dynamic interactions of cellular signals.

Lithium, the classical “gold standard” drug for treating BD, influences multiple points in this complex network, but its precise mechanism and clinical risks coexist, creating a “lithium dilemma”. On one hand, previous studies suggest that lithium’s therapeutic action may be mediated through three primary pathways: it can upregulate AKT protein levels, reduce PIP2 levels by inhibiting IMPase, and directly inhibit GSK3β activity through competitive displacement of magnesium cofactors [29,30,31,32,33]. However, which of these three effects is dominant has long been debated, representing a critical knowledge gap.

On the other hand, lithium treatment is a double-edged sword. It activates the p38 MAP kinase pathway, leading to enhanced NF-κB-dependent transcriptional activity and increased production of pro-inflammatory cytokines such as interleukin-8, thereby promoting an inflammatory response in cells [34,35]. This specific pro-inflammatory mechanism is considered a major reason for the high risk of chronic kidney disease (CKD) and renal function decline in long-term lithium users, creating an urgent need to find safer and more effective alternative therapies.

To address these challenges, this study adopted a systems biology research strategy. We constructed an ordinary differential equation (ODE) computational model that integrates the interaction between the PI3K/AKT pathway and the PI cycle, aiming to achieve two core objectives: (1) to quantitatively deconstruct the triple regulatory mechanism of lithium to identify its primary mode of inhibiting GSK3β; (2) to evaluate the potential of the natural compound curcumin as a novel therapeutic candidate, investigating whether it offers superior efficacy and safety. Through this integrated approach combining computational modeling and molecular docking, we aim to deeply elucidate the molecular basis of BD and T2D comorbidity and to provide a new theoretical basis and candidate solutions for developing more precise and safer treatment strategies.

The simulation results of this study provide clear answers to the aforementioned scientific questions. First, our model reveals that among lithium’s triple action mechanisms, its direct competitive binding to GSK3β is the dominant factor in inhibiting the kinase’s activity, thereby restoring insulin sensitivity. This provides a key mechanistic explanation for its therapeutic effect in the comorbid state of BD and T2D. More importantly, the study found that curcumin is not only a more potent inhibitor of GSK3β than lithium but also exhibits a unique dual advantage: molecular docking analysis confirmed that curcumin can effectively bind to and inhibit p38 MAPK, a key protein in the pro-inflammatory pathway activated by lithium, whereas lithium itself has no such effect. This dual-action property of targeting the core therapeutic target (GSK3β) while also inhibiting a key inflammatory pathway (p38 MAPK) makes curcumin a highly promising drug candidate, potentially offering a safer and more comprehensive solution for the complex clinical challenge of treating BD and T2D comorbidity.

## 2. Results

### 2.1. Constructing and Validating a Computational ODE Model of the BD-T2D Crosstalk Network

**Figure 1:**
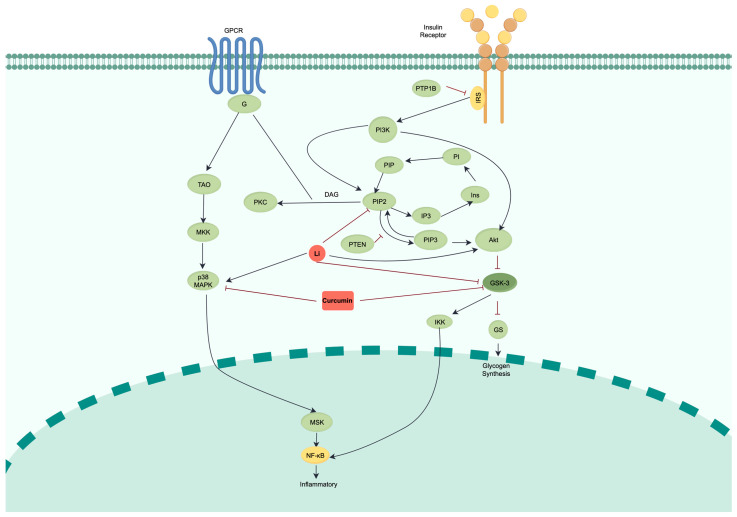
Schematic of the integrated PI3K/AKT, PI cycle, and P38/NF-κB crosstalk network. The schematic illustrates the three interconnected signaling modules incorporated into our ODE-based computational model: (1) Insulin signaling cascade (upper section): Insulin (I) binding to its receptor (IR) initiates the pathway through receptor phosphorylation and IRS recruitment; (2) PI3K/AKT pathway (right section): Activated IRS recruits PI3K, converting PIP2 to PIP3, which subsequently activates AKT. PTEN dephosphorylates PIP3. Activated pAKT phosphorylates and inactivates GSK3β, the primary therapeutic target; (3) PI cycle (middle section): PIP2 hydrolysis by PLC generates IP3 and DAG, which are recycled back to PI through sequential enzymatic reactions involving IMPase. The PI cycle replenishes PIP2, thereby modulating PI3K/AKT signaling intensity. In addition, we add (4) Inflammatory pathway (left section): The p38 MAPK/NF-κB cascade contributes to BD pathophysiology through inflammatory responses. Visual annotations: Arrows indicate the direction of signal flow or biochemical conversion. Brown solid lines represent direct molecular interactions or enzymatic reactions. Red blunt-ended lines (⊣) represent inhibitory interactions. Drug target sites: Lithium (Li) acts at multiple nodes: directly inhibits GSK3β, inhibits IMPase in the PI cycle (reducing PIP2 regeneration), and modulates AKT levels. Curcumin directly inhibits GSK3β and additionally targets the inflammatory mediators p38 MAPK and NF-κB (shown in the left pathway), representing its dual therapeutic advantage over lithium. Abbreviations: IRS, insulin receptor substrate; PI3K, phosphoinositide 3-kinase; PIP2, phosphatidylinositol 4,5-bisphosphate; PIP3, phosphatidylinositol 3,4,5-trisphosphate; PTP1B, protein tyrosine phosphatase 1B; AKT, protein kinase B; GSK3, glycogen synthase kinase 3; PTEN, phosphatase and tensin homolog; IP3, inositol 1,4,5-trisphosphate; Ins, inositol; DAG, diacylglycerol; PI, phosphatidylinositol; PIP, phosphatidylinositol 4-phosphate; PKC, protein kinase C; GS, glycogen synthase; GPCR, G-protein coupled receptor; G, G protein; TAO, thousand-and-one amino acid protein kinase; MKK, mitogen-activated protein kinase; p38 MAPK, p38 mitogen-activated protein kinase; MSK, mitogen- and stress-activated kinase; NF-κB, nuclear factor kappa B; IKK, inhibitor of nuclear factor kappa B kinase.

To investigate the shared mechanisms between BD and T2D, we first collected data on 3605 BD-associated genes and 7594 T2D-associated genes from the GeneCards, OMIM, and TTD databases, identifying 2690 genes that are common to both conditions (Supplementary Table S1). Pathway enrichment analysis revealed a strong association between these shared genes and the PI3K/AKT pathway (Supplementary Table S2), confirming its relevance as a potential mechanistic link. Building upon this, and considering that lithium’s therapeutic effects are hypothesized to involve not only direct inhibition of GSK3β but also inhibition of PI cycle activity, we constructed a comprehensive crosstalk network that integrates both the PI3K/AKT pathway and the PI cycle (Figure 1).

To understand the dynamic behavior of this network and how it responds to therapeutic intervention, we developed a computational model using a system of ODEs to simulate the network’s kinetics. This integrated ODE model encompasses 28 substances and 28 biochemical reactions (Table 1), providing a comprehensive representation of the insulin signaling cascade and its downstream interactions and a robust framework for simulating the complex interplay between these systems. To validate this computational model, we compared its in silico simulated outcomes with previously reported experimental observations from the literature. The temporal dynamics of key protein concentrations and activities generated by our model showed significant correspondence with established reference data [36,37,38,39], confirming the model’s ability to accurately reproduce the biological system’s behavior. This validation step was crucial, as it established our ODE model as a reliable tool for the subsequent in silico experiments designed to dissect therapeutic mechanisms.

### 2.2. Crosstalk Simulation Reveals GSK3β as a Central Regulatory Node for Lithium’s Action

**Figure 2:**
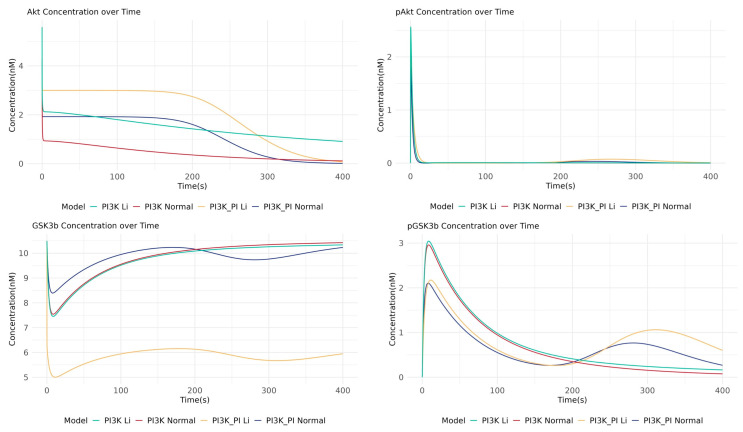
Dynamic responses of AKT and GSK3β to lithium treatment in the PI3K/AKT-only model versus the integrated crosstalk model. The inclusion of the PI cycle crosstalk reveals a more complex, biphasic response in pGSK3β inactivation (e.g., an additional sub-peak around 310 s), which is absent in the PI3K-AKT-only model, highlighting the necessity of an integrated modeling.

To accurately investigate the therapeutic mechanism of lithium, which is known to affect both the PI3K/AKT pathway and the PI cycle, we first assess the necessity of including this crosstalk. We compared simulation results from our integrated crosstalk model against a simpler PI3K/AKT-only model, focusing on critical drug targets in BD such as AKT and GSK3β. The simulation results revealed a critical difference in the dynamics of GSK3β inhibition. The crosstalk model predicted a more complex, biphasic response for inactive pGSK3β, showing an additional sub-peak around 310 s that was absent in the PI3K/AKT-only model (Figure 2). This suggests a synergistic regulatory effect that is only captured when the PI cycle’s influence is included, indicating that the crosstalk network provides a more realistic simulation of the cellular environment.

Having established the crosstalk model as a more faithful representation, we used it to pinpoint the primary molecular target of lithium. We performed a quantitative analysis of the total exposure of active AKT (pAKT) and inactive GSK3β (pGSK3β) under simulated lithium treatment (Table 2). The results clearly distinguished the response of the two nodes. Lithium’s effect on AKT activation was modest; in the crosstalk model, the percentage of active pAKT increased only slightly from 1.88% to 2.35%. In stark contrast, the effect on GSK3β was profound. In the same model, lithium treatment caused the percentage of inactive pGSK3β to more than double, soaring from 6.82% to 14.07%. This striking difference in the magnitude of the response—a minor shift in upstream AKT activation versus a dramatic increase in downstream GSK3β inhibition—provides evidence that GSK3β is the critical and primary regulatory node through which lithium exerts its major therapeutic effect in this network.

### 2.3. Deconvoluting Lithium’s Mechanism Suggests Direct GSK3β Inhibition as the Primary Mode of Action

To quantitatively resolve which of lithium’s three proposed therapeutic pathways is dominant, we then utilized our validated crosstalk model to dissect its precise mechanism of action. We based our quantitative simulations on key findings from previous research that have quantified lithium’s effects on these pathways. First, lithium can enhance the activity of AKT, a serine/threonine kinase that is pivotal in promoting neuronal survival and protecting against apoptosis [46]. Experimental data show that a therapeutic dose of 0.2 μM lithium can increase AKT protein expression by 30% in hippocampal neurons [47]. Second, lithium is known to inhibit inositol monophosphatase (IMPase), reducing PIP2 levels by 37% and thereby influencing the PI cycle [48]. Third, lithium directly competes for a magnesium binding site in GSK3β (with a Ki of approximately 1–2 μM) [20]. To determine which of these three effects contributes most to lithium’s therapeutic effect, we simulated each mechanism individually and in combination. Specifically, the *AKT Modulation* condition simulates a 30% increase in initial AKT concentration; the *PIP2 Modulation* condition simulates a 37% decrease in PIP2; and the *GSK3β-Li Binding* condition simulates the direct competitive inhibition of GSK3β with a Ki of 1 μM.

The simulation results (Table 3 and Supplementary Figure S1) allow for a clear dissection of these mechanisms. In our model, therapeutic efficacy is indicated by the inactivation of GSK3β (pGSK3β), which occurs via phosphorylation by pAKT. The percentage of phosphorylated GSK3β (pGSK3β%), therefore, serves as a key metric. Under baseline *Control* conditions, 6.82% of GSK3β is inactivated through phosphorylation. The *AKT Modulation* condition increases this to 8.67%, while the *PIP2 Modulation* has a negligible effect (6.73%). The most striking result is seen in the *GSK3β-Li Binding* condition. Here, even as lithium directly binds to and inhibits a portion of the total GSK3β pool, the proportion of the remaining GSK3β that becomes inactivated via phosphorylation rises dramatically to 11.33%. Our simulation suggests that direct binding makes the remaining GSK3β population significantly more susceptible to upstream inactivation by AKT. Since this percentage (11.33%) is substantially higher than that achieved by modulating AKT alone (8.67%) and is very close to the result when all effects are combined (*Total Effects*, 14.07%), it confirms that the direct binding and inhibition of GSK3β is the dominant therapeutic mechanism of lithium in this system.

### 2.4. Curcumin vs. Lithium Effects on GSK3β and Related Pathways

While our model confirms lithium’s efficacy in inhibiting its primary target, GSK3β, its clinical use is hampered by significant side effects. Notably, lithium activates the p38 MAP kinase pathway, leading to enhanced NF-κB-dependent transcriptional activity and increased production of pro-inflammatory cytokines such as interleukin-8, thereby promoting an inflammatory response in cells [34,35]. This inflammatory effect may contribute to the high risk of CKD and renal function decline observed in long-term lithium users. This motivated the search for a safer alternative that retains or exceeds the therapeutic benefit.

**Figure 3:**
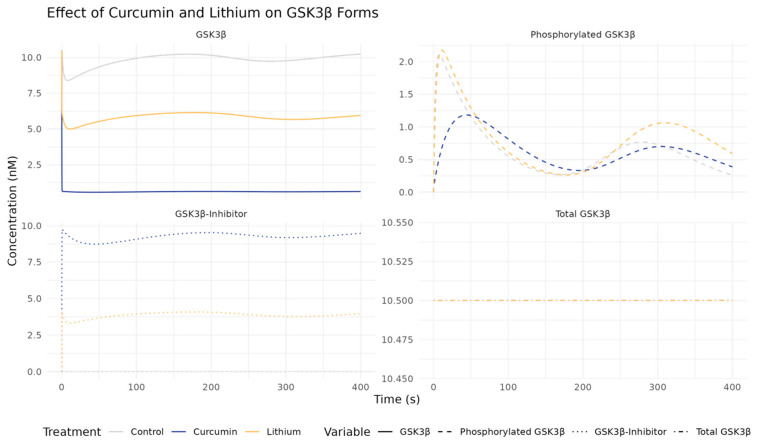
Comparative effects of curcumin (1000 nM) and lithium (1000 nM) on the dynamics of key signaling molecules in the crosstalk model. Simulations indicate that curcumin treatment results in lower levels of active GSK3β and higher levels of its inactive form (pGSK3β) compared to lithium, suggesting superior inhibitory efficacy.

We investigated curcumin, a natural compound known to target GSK3β through direct binding with a lower IC50 of 66.3 nM, compared to lithium’s higher IC50 of 1–2 μM. Curcumin has been found potential in regulating issues related to neurodegeneration and mental disorders [49] and enhancing insulin sensitivity in insulin-resistant HepG2 cells by boosting PI3K-AKT-GSK3β signaling and inhibiting ERK/JNK phosphorylation [50]. These collectively suggest curcumin’s dual role in improving mood regulation and metabolic health, making it a potential candidate for both BD and T2D treatment. Thus, we first compared its efficacy directly against lithium in our crosstalk model. The kinetic rate constants (K_on_ and K_off_) for curcumin were estimated based on the IC50 values against GSK3β from in vivo studies [51]. As a result, our simulations showed that curcumin, at the same concentration (1000 nM), outperformed lithium in inhibiting GSK3β, as evidenced by lower total GSK3β levels and higher levels of its inactive form pGSK3β (Figure 3).

**Figure 4:**
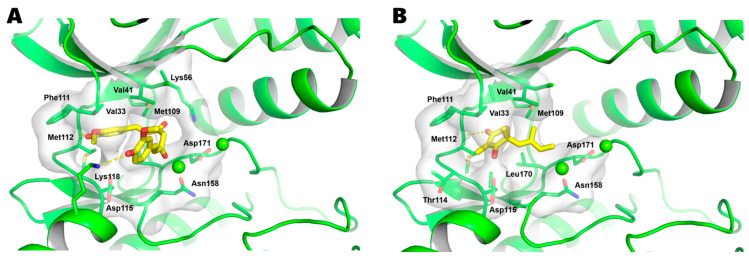
Molecular docking analysis reveals curcumin’s ATP-competitive binding mode within the p38 MAPK catalytic site. The p38 MAPK ATP-binding pocket, located in the deep cleft between the N- and C-terminal domains, serves as the enzyme’s catalytic core and is a critical target for regulating inflammatory responses. This analysis compares the predicted binding poses of curcumin and the reference inhibitor SM-101 within this functionally significant site. (**A**) Curcumin (binding energy: −7.6 kcal/mol) is predicted to anchor within the active site through two key hydrogen bonds (indicated by dashed lines): one with the backbone of the conserved hinge residue Met112 and another with the side chain of Lys118. The interaction with the hinge region residue Met112 is a hallmark of Type I ATP-competitive inhibitors, effectively mimicking the binding of the natural substrate ATP. Furthermore, curcumin’s aromatic rings are stabilized by extensive hydrophobic interactions with key residues lining the pocket, including Val33, Val41, Lys56, and Phe111. (**B**) For comparison, the reference inhibitor SM-101 (binding energy: −6.1 kcal/mol) adopts a similar orientation, likewise forming the crucial hydrogen bond with Met112 and engaging in hydrophobic contacts with Val33, Val41, and Phe111. The significant overlap in their binding poses, particularly the shared, critical hydrogen bond with the hinge residue Met112, provides strong structural evidence that curcumin inhibits p38 MAPK through an ATP-competitive mechanism.

To investigate its effect on the inflammatory pathways activated by lithium, we then performed molecular docking simulations targeting the key inflammatory protein p38 MAPK. As shown in Figure 4, curcumin binds favorably within the ATP-binding pocket of p38 MAPK, achieving a strong binding energy of −7.6 kcal/mol, which is superior to that of the reference inhibitor SM-101 (−6.1 kcal/mol). A detailed analysis of the binding pose revealed that curcumin is anchored by two key hydrogen bonds: one with the backbone of the conserved hinge residue Met112 and another with the side chain of Lys118. Additionally, curcumin’s aromatic structure is further stabilized by extensive hydrophobic interactions with Val33, Val41, Lys56, and Phe111. Notably, curcumin’s binding mode shows significant overlap with that of SM-101, as both share the critical hydrogen bond with Met112. This shared interaction strongly suggests that curcumin exerts its effect via an ATP-competitive mechanism. Further analysis showed curcumin also binds effectively to the inflammatory transcription factor NF-κB (−7.0 kcal/mol, Supplementary Figure S2).

These findings collectively suggest that curcumin may be a more potent inhibitor of the primary therapeutic target, GSK3β, but also possesses a significant anti-inflammatory advantage by directly engaging key inflammatory mediators. This multi-target action makes curcumin a highly promising therapeutic candidate for managing both BD and T2D, where inflammation and metabolic dysregulation are intertwined.

## 3. Discussion

Recent network-based analyses have highlighted the PI3K/AKT pathway as a potential shared mechanism linking T2D and neuropsychiatric disorders, including BD [12]. Our pathway enrichment analysis of shared genes between BD and T2D independently corroborated this link, providing a critical empirical foundation for the present study. Building on this validation, we developed a quantitative computational model to move beyond static pathway identification and investigate the dynamic regulatory principles governing this crucial signaling axis. More importantly, our integrated model unites the PI3K/AKT pathway with the PI cycle, thereby merging two major hypotheses of BD pathology into a single cohesive framework. This comprehensive model, constructed using ODEs, captures the complex interplay between these pathways via PIP2, providing a more detailed representation of cellular signaling dynamics. Comparative simulations with or without the PI cycle demonstrate the necessity of integrating multiple signaling pathways and kinetic reactions to accurately model BD pathophysiology.

Our study also presented a computational approach to quantitatively dissect lithium’s regulatory effects. Lithium is known to influence multiple components of the signaling network by increasing AKT levels, reducing PIP2 levels, and directly binding to GSK3β [20,52]. We validated the model by simulating several key components, with outcomes showing consistency with previous experimental observations. While lithium’s direct binding to GSK3β has been well established [20,52], the relative contributions of these three regulatory mechanisms remained unclear. Importantly, our quantitative analysis demonstrated that GSK3β inhibition is largely driven by lithium’s direct binding rather than by indirect regulatory effects, providing a quantitative framework for understanding the relative importance of lithium’s multiple mechanisms of action within this signaling network.

While lithium remains an effective treatment for BD, its prolonged use raises concerns about renal health, including an increased risk of CKD. Lithium inhibits GSK3 activity by targeting the PI3K/AKT pathway and enhancing extracellular-regulated pathway signaling (p38 MAPK/NFκB). These alterations reduce renal response to vasopressin, contributing to chronic interstitial renal disease and CKD through inflammation, oxidative stress, and fibrosis [53]. Additionally, the activation of the p38-NFκB axis by lithium induces an inflammatory response, exacerbating renal damage. Given this, anti-inflammatory agents could offer protective effects in cases of kidney injury, highlighting their potential role in managing lithium-induced renal complications.

In contrast, our computational analysis predicts a key mechanistic advantage for curcumin over lithium, stemming from its multi-target engagement profile. In addition to its potent inhibition of GSK3β, our molecular docking simulations revealed that curcumin binds effectively to the pro-inflammatory mediators p38 MAPK and NFκB. This finding is of particular significance for the treatment of BD-T2D comorbidity, as the pathological roles of both p38 MAPK and NFκB in T2D are well-established [54,55,56,57,58]. These pathways are central drivers of the chronic, low-grade inflammation that promotes and exacerbates insulin resistance. Therefore, curcumin’s predicted ability to simultaneously inhibit GSK3β—a core target in both BD and insulin signaling—and suppress the p38 MAPK/NFκB inflammatory axis suggests it may offer a more integrated therapeutic strategy, addressing both the neuro-regulatory and metabolic dysfunctions inherent to the comorbidity. It is critical, however, to acknowledge that this compelling mechanistic hypothesis is derived solely from our computational modeling and molecular docking studies, and thus awaits experimental validation in biological systems.

A critical consideration for the clinical translation of curcumin in treating central nervous system (CNS) disorders like BD is its well-documented pharmacokinetic limitations. In its native form, curcumin exhibits poor aqueous solubility, rapid systemic metabolism, and consequently, extremely low oral bioavailability (<1%), resulting in negligible brain tissue concentrations (e.g., 1.4 ± 0.8 ng/g) [59]. This stands in stark contrast to lithium, which readily achieves therapeutic brain concentrations (0.47–0.9 mM) via conventional oral administration [60]. This significant barrier has historically limited the therapeutic application of curcumin for neurological and psychiatric conditions, rendering any theoretical benefits difficult to realize in clinical practice. However, recent advancements in pharmaceutical formulation technology are fundamentally changing this landscape. Innovative drug delivery systems have been developed to substantially overcome curcumin’s bioavailability and blood–brain barrier (BBB) penetration challenges. For instance, a novel curcumin-galactomannoside formulation has been shown to achieve brain tissue concentrations of 343 ± 64.7 ng/g (~930 nM) in preclinical models—representing a 245-fold enhancement in BBB permeability compared to standard curcumin [59]. Similarly, other strategies employing PLGA nanoparticles and lipid-based carriers have demonstrated up to 30-fold enhanced brain uptake [61,62]. Meanwhile, the brain concentration achieved by the curcumin-galactomannoside formulation (~930 nM) remarkably approaches the 1000 nM concentration used in our ODE simulations, indicating that the dual GSK3β/p38 MAPK inhibition predicted by our model is potentially achievable.

While clinical translation for BD is currently limited by a lack of dedicated trials, our study provides a strong, mechanism-based argument for prioritizing such research. By demonstrating curcumin’s theoretical multi-target advantages at concentrations that are becoming increasingly attainable in vivo, our work offers a rational basis for advancing specific, optimized curcumin formulations into preclinical and clinical investigations for BD-T2D comorbidity. Future studies should focus on validating these predicted mechanisms and establishing optimal dosing strategies for these novel formulations in relevant patient populations.

Nonetheless, our study has several important limitations that also highlight promising avenues for future research. First, our conclusions regarding curcumin’s dual-target efficacy are entirely computational and require rigorous experimental validation in cell-based assays, animal models, and/or clinical studies. Second, while our discussion incorporates achievable brain concentrations from advanced formulations, the current ODE model does not simulate dynamic pharmacokinetic parameters such as absorption rates, clearance kinetics, or dose–response relationships. Future multi-scale models should integrate these parameters to translate our mechanistic predictions into optimized dosing strategies. Third, the model’s quantitative accuracy is constrained by the availability of kinetic parameters in the literature; further experimental measurements would enhance its predictive power. Finally, our current model is limited to the PI3K/AKT pathway and PI cycle, excluding other critical systems such as p38 MAPK, mTOR, Wnt/β-catenin that are also implicated in BD and T2D. This simplification, while necessary for computational tractability, does not capture the full complexity of shared pathophysiological mechanisms. Future iterations should integrate additional signaling networks to better represent the multifactorial nature of BD-T2D comorbidity.

In conclusion, this study makes two primary contributions to understanding and potentially treating the comorbidity of BD and T2D. First, through a quantitative, integrated computational model, we deconvoluted the complex mechanisms of lithium, demonstrating that its therapeutic effect is predominantly driven by the direct inhibition of GSK3β. Second, our analysis identified curcumin as a highly promising multi-target alternative. We provide a compelling theoretical basis for its dual action: inhibiting the core therapeutic target GSK3β while simultaneously suppressing the pro-inflammatory p38 MAPK/NFκB axis, a key driver of insulin resistance. Crucially, we show that the concentrations required for this dual effect are becoming pharmacologically achievable with advanced drug delivery systems. Together, these findings offer novel mechanistic insights and provide a strong, data-driven rationale for prioritizing the clinical investigation of next-generation curcumin formulations for this challenging comorbidity.

## 4. Materials and Methods

### 4.1. Data Collection

BD- and T2D-related genes were collected from Genecard, OMIM, and TTD databases, respectively. GeneCards with a relevance score of no less than 10. Duplicated genes were removed. All genes associated with BD and T2D were analyzed using Venn diagrams to identify potential shared genes between the two conditions.

### 4.2. Pathway Enrichment Analysis

To identify significant biological pathways related to BD and T2D, we performed Kyoto Encyclopedia of Genes and Genomes (KEGG) enrichment analysis. This was performed using the clusterProfiler 4.2.0, with gene identifiers in Entrez format. The enrichment analysis was carried out using a *p*-value cutoff of 0.05 to determine statistically significant pathways.

### 4.3. ODE Simulation

In this study, we used ordinary differential equations (ODEs) to construct a dynamic model of the PI3K/AKT signaling pathway and its interaction with the phosphoinositide (PI) cycle under lithium treatment. This modeling approach allowed us to simulate both mass action kinetics and enzyme-catalyzed reactions to capture the complexities of intracellular signaling dynamics. The reaction components and their initial values are detailed in Supplementary Table S3. Additionally, the molecular interactions and their corresponding kinetic constants are comprehensively described in Table 1. ODEs were formulated to represent these interactions based on the law of mass action, with rate constants determined by forward (Kf) and reverse (Kb) reactions from publications. For enzyme-catalyzed processes, turnover numbers (Km) were sourced from previously published models.

Lithium concentration was set at 1000 nM (1 μM) based on in vitro neuronal studies demonstrating GSK3β inhibition at sub-therapeutic to therapeutic concentrations (0.02–2 mM) [47]. We selected 1 μM as it falls within the lower end of this range and is appropriate for our ODE system, where baseline protein concentrations range from 0.0009 nM to 300 nM (Table 1). This concentration enables mechanistic investigation of direct GSK3β inhibition while remaining computationally tractable within our network scale. Although clinical therapeutic serum lithium concentrations for BD treatment range from 0.6 to 1.2 mM [60], our model focuses on the molecular mechanism of GSK3β inhibition rather than replicating clinical pharmacokinetics. For curcumin, we used 1000 nM to enable direct 1:1 molar comparison with lithium. This concentration is physiologically achievable: a curcumin-galactomannoside formulation achieved brain tissue concentrations of 343 ± 64.7 ng/g (~930 nM) in rats, representing a 245-fold improvement over native curcumin [59]. This demonstrates that our simulation concentration, while facilitating mechanistic comparison, is attainable with optimized delivery systems.

### 4.4. Mass Action Kinetics

For general biochemical reactions, we modeled the rate of change in concentration based on the law of mass action, which states that the reaction rate is proportional to the product of the concentrations of the reactants. The basic form of these biochemical interactions is represented as follows:

$$\alpha A+\beta B\overset{k}{\to }\gamma C$$

In this equation, A and B are reactants, C is the product, and α, β, and γ denote stoichiometric coefficients, while k is the reaction rate constant. The rate of the reaction can be expressed as:

$$\frac{d[C]}{dt}=k{\left[A\right]}^{\alpha }{\left[B\right]}^{\beta }$$

In the ODE model, both forward and reverse reactions were considered, as molecules in the biochemical system are in constant random motion. The forward rate constant is denoted as $K_{on}$ (or $K_{f}$), while the reverse rate constant is denoted as $K_{off}$ (or $K_{b}$), which are calculated based on equilibrium relationships:

$$K_{on}=\frac{{\left[C\right]}^{\gamma }}{{\left[A\right]}^{\alpha }{\left[B\right]}^{\beta }}$$

$$K_{off}=\frac{{\left[A\right]}^{\alpha }{\left[B\right]}^{\beta }}{{\left[C\right]}^{\gamma }}$$

Depending on the nature of the reaction, we considered reactions as either reversible or irreversible. Reversible reactions were represented by an equal sign (**=**), whereas irreversible reactions were represented by an arrow (**→**). For irreversible reactions, the reverse rate constant ($K_{b}$) was considered negligible due to its minimal effect on reaction dynamics.

### 4.5. Enzyme-Catalyzed Reactions

In addition to mass action kinetics, we modeled enzyme-catalyzed reactions using Michaelis–Menten kinetics to accurately describe the enzyme–substrate dynamics in the PI3K/AKT pathway. Enzyme-catalyzed reactions differ from simple mass action reactions due to the involvement of an enzyme that facilitates the conversion of substrate to product. The general form of an enzyme-catalyzed reaction is represented as:

$$E+S\overset{K_{1}}{\to }ES\overset{K_{2}}{\to }E+P$$

where E is the enzyme, S is the substrate, ES is the enzyme–substrate complex, and P is the product. The rate of the reaction can be described using the Michaelis–Menten equation:

$$\frac{d\left[P\right]}{dt}=\frac{V_{max}\left[S\right]}{K_{m}+\left[S\right]}$$

where $V_{max}$ resents the maximum rate of the reaction and $K_{m}$ (Michaelis constant) is the substrate concentration at which the reaction rate is half of $V_{max}$. This modeling allowed us to account for saturable dynamics typical in enzyme–substrate interactions, providing a more accurate representation of reactions mediated by enzymes such as kinases within the PI3K/AKT pathway.

### 4.6. Incorporating Inhibition in the ODE Model Using IC50

To incorporate the effects of inhibitors, particularly on Enzyme E, we used a mass action kinetics approach to model the binding of the inhibitor to its target. The binding reaction is represented by:

E+Inhibitor⇌E−Inhibitor

The rate of this reaction is described by:

$$\frac{d\left[E-Inhibitor\right]}{dt}=k_{on}\left[E\right]\left[Inhibitor\right]-k_{off}[E-Inhibitor]$$

where $k_{on}$ is the association rate constant and $k_{off}$ is the dissociation rate constant. The IC50 value represents the concentration of inhibitor required to achieve 50% inhibition.

The relative activity of GSK3β is calculated as:

$$Relative\;Activity\;\left(\%\right)=\left(\frac{\left[E\right]}{\left[Total\;E\right]}\right)\times 100$$

where $\left[Total\;E\right]$ the sum of free GSK3β, phosphorylated GSK3β, and inhibitor-bound GSK3β at the initial time point. This approach allows for a mechanistic representation of the inhibition process, taking into account the dynamic binding and unbinding of the inhibitor to Enzyme E, and provides a quantitative measure of enzyme activity under various inhibitor concentrations.

### 4.7. ODE Implementation in R

To implement the ODE simulation, we used the R version 4.3.2. The deSolve package 1.40 was employed to numerically solve the system of differential equations with method ‘lsoda’, allowing us to simulate both mass action and enzyme-catalyzed reactions effectively. For data visualization, ggplot2 4.0.0 was used to illustrate the time course of pathway component concentrations, while igraph 1.2.8 was utilized to visualize the network structure of signaling interactions.

### 4.8. Molecular Docking

To analyze the binding affinities and interaction modes between drug candidates and their targets, molecular docking simulations were performed using AutoDock Vina 1.2.2. The molecular structures of lithium (CID: 3028194), curcumin (CID: 969516) and SM-101 (CID: 135403829) were retrieved from PubChem, while the 3D coordinates of the target proteins p38 MAPK (PDB ID: 1CM8), NF-kB (PDB ID: 1NFK) were obtained from the Protein Data Bank. Protein and ligand files were converted to PDBQT format, excluding water molecules and adding polar hydrogens. Docking simulations were conducted within a grid box of 30 Å × 30 Å × 30 Å, with a grid point distance of 0.05 nm. The resulting PDB file was then analyzed using the Protein–Ligand Interaction Profiler to assess binding interactions.

## Figures and Tables

**Table 1:** Chemical reactions and kinetic parameters in the integrated PI3K/AKT and PI cycle crosstalk network.

	Chemical Reactions	K_f_ (nM^−1^·s^−1^ or s^−1^)	K_b_ (nM^−1^·s^−1^ or s^−1^)	References
1	I + IR = IRL	k_1_1_: 3.33 × 10^−4^	k_1_2_: 1	[40]
2	IRL -> IRp	k_2_: 1.66	-	[40]
3	IRp -> IRi	v = V_max_ × IRp/(K_m_ + IRp), V_max_ = 333 nM^−1^s^−1^, K_m_ = 266 nM		[40]
4	IRi -> IRL	k_4_: 0.0166	-	[40]
5	IRp -> Null	k_5_: 1.85 × 10^−4^	-	[41]
6	IRp + IRS = IRp-IRS	k_6_1_: 0.1066	k_6_2_: 3.75	[40]
7	IRp-IRS -> IRS	k_7_: 1.85 × 10^−4^	-	[41]
8	IRp-IRS -> IRp-IRSp	k_8_: 0.66	-	[40]
9	IRp-IRSp + PTP1B -> IRp-IRS	k_9_: 8.75 × 10^−5^	-	[37]
10	IRp-IRSp -> Null	k_10_: 0.001	-	[37]
11	IRp-IRSp + PI3K = IRSp-PI3K	k_11_1_: 2.6 × 10^−6^ *	k_11_2_: 1.55 *	[37]
12	IRSp-PI3K + PIP2 -> PIP3	k_12_: 0.055	-	[37,41]
13	PIP3 + PTEN -> PIP2	k_13_: 0.7025	-	[41]
14	AKT + PIP3 -> pAKT	k_14_: 3.36	-	[38]
15	pAKT -> AKT	k_15_: 1.188 × 10^−6^	-	[37]
16	ppAKT + GSK3β -> pGSK3β	k_16_: 0.05	-	[36]
17	pGSK3β -> GSK3β	k_17_: 0.015	-	[36]
18	PIP2 -> IP3 + DAG	k_18_: 500.25	-	[42]
19	IP3 -> Ins	k_19_: 10	-	[43]
20	Ins -> PI	k_20_: 0.7081	-	[42]
21	PI = PIP	k_21_1_: 0.4		[44]
22	PIP = PIP2	k_22_1_: 60	k_22_2_: 5	[44]
23	G = G-a	k_23_1_: 5.18 × 10^−6^	k_23_2_: 0.085	[42]
24	PIP2 + G-a -> DAG	k_24_: 77.16	-	[42]
25	DAG -> PIP2	k_25_: 0.0077	-	[42]
26	DAG + PKC -> PKC-a	k_26_: 1.311	-	[42]
27	PKC-a -> PKC	k_27_: 0.0295	-	[42]
28	PIP3 -> Null	3.33		[45]

Notes: “Null” indicates the absence of any variable (species). The reaction rate of IRp -> IRi is not a mass action but involves the Michaelis–Menten equation, which is a single-substrate Michaelis–Menten equation. * Estimated kinetic data.

**Table 2:** Total exposure (concentration–time integral, nM·s) of AKT and GSK3β in the PI3K/AKT-PI crosstalk model and the PI3K/AKT-only model under lithium treatment.

	PI3K_PI Normal	PI3K_PI Li	PI3K Normal	PI3K Li
AKT	473.58	818.24	162.00	571.93
pAKT	8.92	19.26	9.37	10.48
pAKT%	1.88%	2.35%	5.78%	1.83%
GSK3β	3931.82	2327.35	3924.94	3896.44
pGSK3β	268.18	327.48	275.06	303.57
pGSK3β%	6.82%	14.07%	7.01%	7.79%

Note: PI3K_PI Normal—the integrated model without treatment; PI3K_PI Li—the integrated model simulated under lithium treatment; PI3K Normal—the PI3K/AKT pathway-only model without treatment; PI3K Li—the PI3K/AKT pathway-only model under lithium treatment.

**Table 3:** Total exposure (concentration–time integral, nM·s) of AKT and GSK3β under individual and combined effects of lithium’s mechanisms of action.

	Control	AKT Modulation	PIP2 Modulation	GSK3β-Li Binding	Total Effects
GSK3β-Li Complex	0.00	0.00	0.00	1568.92	1545.16
AKT	473.58	782.62	496.51	473.58	818.24
pAKT	8.92	11.66	8.90	14.82	19.26
pAKT%	1.88%	1.49%	1.79%	1.88%	2.35%
GSK3β	3931.82	3865.00	3935.19	2363.27	2327.35
pGSK3β	268.18	335.00	264.81	267.81	327.48
pGSK3β%	6.82%	8.67%	6.73%	11.33%	14.07%

Control: Baseline condition without lithium treatment. AKT Modulation: 30% increase in AKT concentration. PIP2 Modulation: 37% decrease in PIP2 concentration. GSK3β-Li Binding: Formation of GSK3β-lithium complex. Total Effects: Simultaneous application of all above modulations.

## Data Availability

The data underlying this article are available in the article and in its online Supplementary Material.

## Supplementary Materials

The following supporting information can be downloaded at: https://www.mdpi.com/article/10.3390/ijms262010026/s1.
