# Supplementary material for: Computational Modeling of PI3K/AKT Pathway in Bipolar Disorder and Type 2 Diabetes: Implications for Lithium Treatment and Curcumin as a Potential Alternative
Source: Int J Mol Sci. 2025 Oct 15;26(20):10026. doi: 10.3390/ijms262010026 (PMC12564203; doi:10.3390/ijms262010026)
Supplement: Supplementary file 1 [file ijms-26-10026-s001.zip › Supplementary Figure S1_S2 & Table S3.pdf]

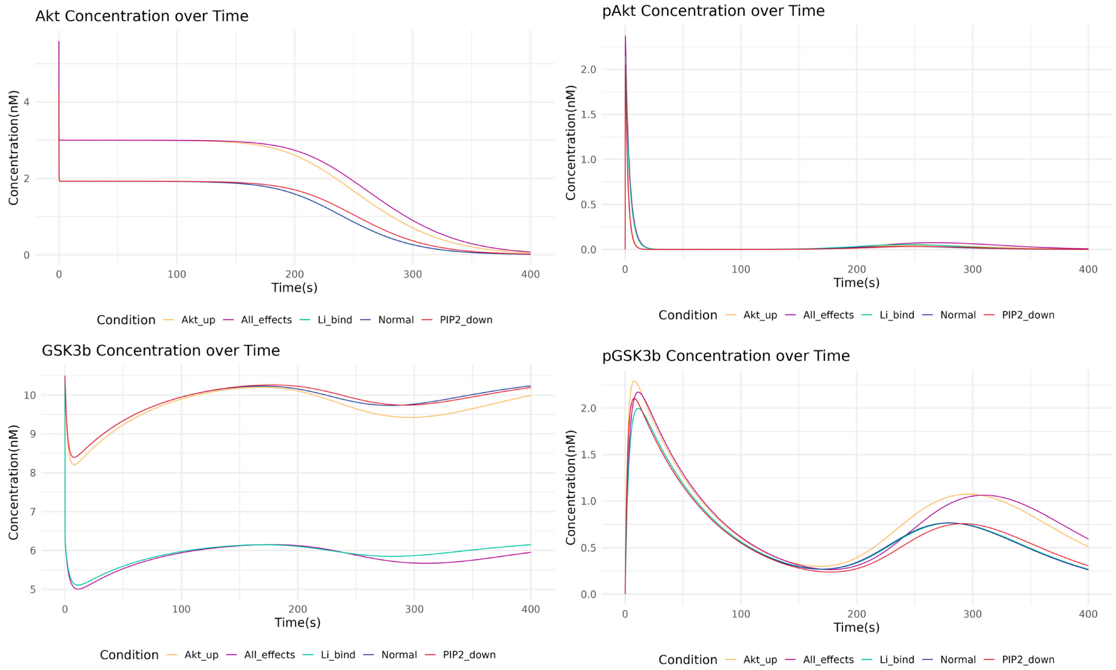

Supplementary Figure S1. Simulated effects of lithium's individual and combined mechanisms on the crosstalk network. Simulation conditions: (1) *AKT modulation* (Akt_up, yellow) - 30% increase in initial AKT to simulate lithium's enhancement of AKT activity; (2) *PIP2 modulation* (PIP2_down, red) - 37% decrease in initial PIP2 to represent lithium's inhibition of IMPase and subsequent reduction in PIP2 synthesis; (3) *GSK3β-Li Binding* (Li_bind, green) – direct inhibition of GSK3β (Ki = 1000 nM); (4) *Total Effects* (All_effects, purple) – all mechanisms combined. Baseline is shown in blue for comparison.

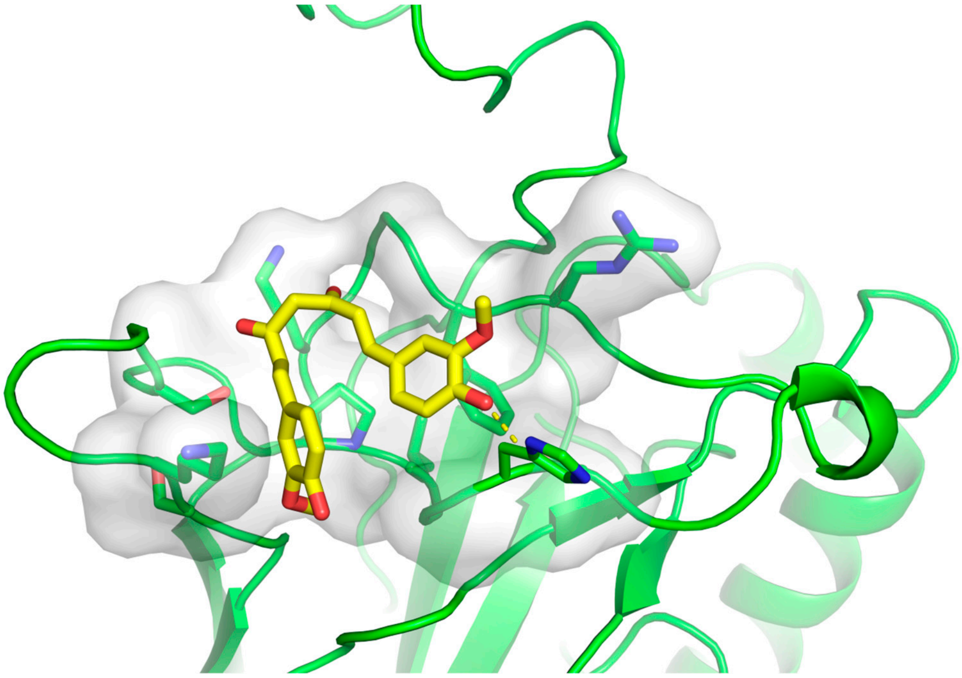

Supplementary Figure S2. Molecular docking result of curcumin bound to the NF-κB binding pocket. Curcumin demonstrates a strong binding affinity for NF-κB, with a calculated binding energy of -7.0 kcal/mol. Hydrogen bonds are represented by dashed lines.

Supplementary Table S3. Reaction substances and initial concentrations in the crosstalk network.

ID	Component	Concentration (nM)	References
y1	I	45	[24]
y2	IR	150	[28]
y3	IRL	0	-
y4	IRp	$8.97*10^{-4}$	[29]
y5	IRi	0	-
y6	IRS	300	[28]
y7	IRp-IRS	0	-
y8	IRp-IRSp	0	-
y9	PTP1B	1	[29]
y10	PI3K	200	[28]
y11	IRSp-PI3K	7	*
y12	PIP2	11	[30]
y13	PIP3	3.332	[30]
y14	PTEN	1	[28]
y15	AKT	4.3	[32]
y16	pAKT	0	-
y17	GSK3β	10.5	[24]
y18	pGSK3β	0	-
y19	IP3	0	-
y20	DAG	0	-
y21	Ins	0	-
y22	PI	0	-
y23	PIP	0	-
y24	PKC	2	[30]
y25	PKC_a	0	-
y26	G	10	-
y27	G_a	0	-

Abbrevations: Insulin (I), Insulin Receptor (IR), Insulin Receptor-Ligand complex (IRL), Phosphorylated Insulin Receptor (IRp), Insulin Receptor Substrate (IRS), Phosphorylated Insulin Receptor-IRS complex (IRp_IRS), Phosphorylated Insulin Receptor-Phosphorylated IRS complex (IRp_IRSp), Protein Tyrosine Phosphatase 1B (PTP1B), Phosphatidylinositol 3-kinase (PI3K), Phosphorylated IRS-PI3K complex (IRSp_PI3K), Phosphatidylinositol 4,5-bisphosphate (PIP2), Phosphatidylinositol 3,4,5-trisphosphate (PIP3), Phosphatase and tensin homolog (PTEN), Protein Kinase B (AKT), Phosphorylated Protein Kinase B (pAKT), Glycogen Synthase Kinase 3 Beta (GSK3b), Phosphorylated Glycogen Synthase Kinase 3 Beta (pGSK3b), Inositol 1,4,5-trisphosphate (IP3), Diacylglycerol (DAG), Inositol (Ins), Phosphatidylinositol (PI), Phosphatidylinositol 4-phosphate (PIP), Protein Kinase C (PKC), Activated Protein Kinase C (PKC_a), G protein (G), Activated G protein (G_a).
